# Platelet-rich plasma loaded nerve guidance conduit as implantable biocompatible materials for recurrent laryngeal nerve regeneration

**DOI:** 10.1038/s41536-022-00239-2

**Published:** 2022-09-14

**Authors:** Ji Won Kim, Jeong Mi Kim, Mi Eun Choi, Eun Jeong Jeon, Jin-Mi Park, Young-Mo Kim, Seung-Ho Choi, Taesik Eom, Bong Sup Shim, Jeong-Seok Choi

**Affiliations:** 1 Department of Otorhinolaryngology-Head and Neck Surgery, Inha University College of Medicine, 27 Inhang-ro, Jung-gu, Incheon, 22332 Republic of Korea; 2 Department of Biomedical Science, Program in Biomedical Science & Engineering, Inha University, 100 Inharo, Michuholgu, Incheon, 22212 Republic of Korea; 3 Department of Otolaryngology, Asan Medical Center, University of Ulsan College of Medicine, 05505 Seoul, Republic of Korea; 4 Department of Chemical Engineering, Inha University, 22212 Incheon, Republic of Korea; 5 Research Center for Controlling Intercellular Communication (RCIC), College of Medicine, Inha University, 100 Inharo, Michuholgu, Incheon, 22212 Republic of Korea

**Keywords:** Spinal cord injury, Regenerative medicine

## Abstract

Vocal cord paralysis caused by recurrent laryngeal nerve (RLN) injury during thyroidectomy results in hoarseness, aspiration, and dyspnea. We evaluated the usefulness of nerve guidance conduits (NGCs) constructed from an asymmetric polycaprolactone (PCL)/Pluronic F127 porous membrane and filled with platelet-rich plasma (PRP) for functional RLN regeneration. We evaluated the proliferation and migration of Schwann cells (SCs) after PRP treatment in vitro. For the in vivo study, rabbits were divided into a non-loaded NGC group and a PRP-loaded NGC group. The left RLNs were resected and interposed with the NGCs. Functional and histological examinations of the vocal cords were performed. SC proliferation and migration increased in a PRP dose-dependent manner, with the PRP increasing the levels of neurotrophic factors, myelin-associated glycoprotein, and ERK. In vivo, the PRP group showed significantly better vocal cord mobility and less vocalis muscle atrophy than the non-loaded NGC group. Histologically, the ingrowth of nerve endings occurred more rapidly in the PRP group, and acetylcholinesterase, neurofilament, and S-100 expression in neural endings were significantly higher in the PRP group. Furthermore, transmission electron microscopy showed that myelinated axons were more tightly packed in the PRP group. This study shows that PRP-loaded NGCs provide a favorable environment for neural regeneration and suggests that this technique has therapeutic potential for promoting RLN recovery.

## Introduction

During thyroid and thoracic surgery, recurrent laryngeal nerve (RLN) injury can result in vocal cord paralysis (VCP)^1^, which manifests as hoarseness, aspiration, and dyspnea and may even be life-threatening^2^. Therefore, when an RLN is invaded by a tumor or injured during surgery, intraoperative reconstruction of the RLN is attempted^3^. Surgical options include (1) RLN primary repair (end-to-end epineural suture) and (2) nerve transplantation, such as free nerve grafting or end-to-side anastomosis^4,5^. However, primary repair is only applicable in cases with RLN defect lengths of ≤5 mm and in both stumps without tension. Furthermore, in the case of nerve transplantation, donor site morbidities are inevitable^6^. Furthermore, misdirected regeneration among nerve fibers is possible, and the vocalis muscles may gradually atrophy during neural regeneration^2,7^. Thus, more effective means are required for functional recovery of the vocal cords.

Regeneration after peripheral nervous system (PNS) injury has better outcomes than that after central nervous system (CNS) injury. The mammalian PNS is capable of axonal outgrowth, which means that substantial functional recovery can occur, while CNS injury results in glial scar formation that inhibits neural growth^8^. Several studies have addressed the optimal environments for peripheral nerve regeneration^9–13^. The most commonly studied methods involve stem cells, growth factors (GFs) containing or eluting materials that stimulate regenerating axons, and implanting nerve guidance conduits (NGCs) and scaffolds at the site of injury^6,14,15^.

In a previous study, NGCs manufactured with an asymmetric polycaprolactone (PCL)/Pluronic F127 porous membrane containing pores of different sizes on the inner (nano-sized) and outer (micro-sized) surfaces were used as implantable biomaterials for the regeneration of rat sciatic nerve^16^. The selective permeability of this membrane prevents the infiltration of inflammatory cells and myofibroblasts, but permits the diffusion of nutrients and oxygen, which are prerequisites for effective nerve revitalization through an NGC^17^. The efficacy of this system was previously demonstrated in an RLN defect model^2^ as well as in a rat sciatic nerve defect model^16^. Recently, a tissue-engineered conduit in combination with neurotrophic factors, GFs, or stem cell release has been used to promote axonal sprouting to increase the regenerative effects^9,16^. On the other hand, platelet-rich plasma (PRP) has been reported to have healing effects on tendons, ligaments, muscle, bone, and on the regeneration of peripheral nerves, which have been ascribed to the various GFs it contains^18,19^. Although the effects of these GFs on nerve regeneration have been studied^20^, little is known about the biological effects of PRP on Schwann cells (SCs)^17,21^.

**Fig. 1:**
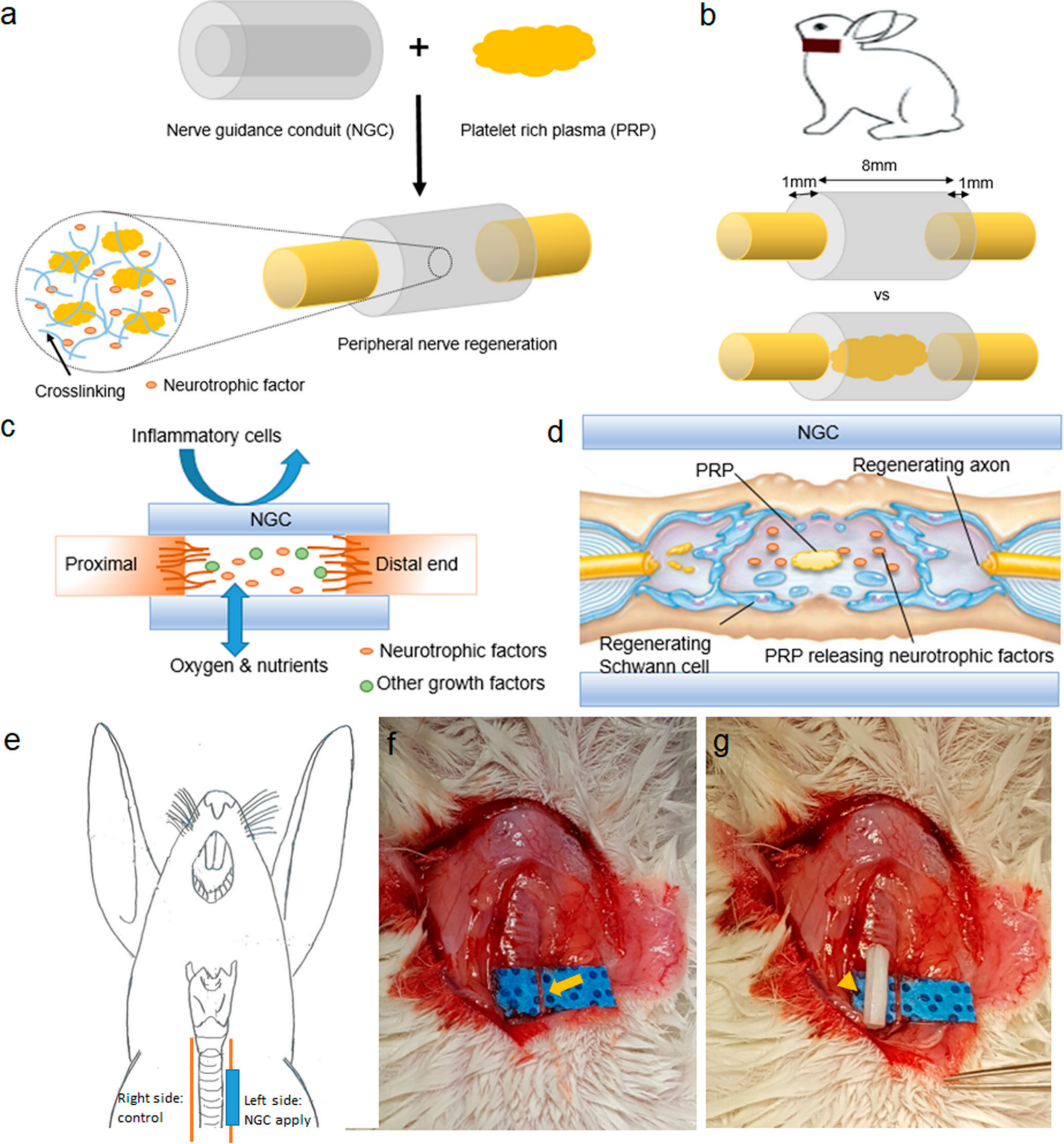
Schematic illustrations of the NGC filled with PRP and application in animal model. **a** NGC was filled with PRP, and activated PRP released growth factors, including neurotrophic factors. **b** RLNs of rabbits were used to compare the efficacies of unfilled *vs* PRP-filled NGCs. **c** Asymmetrically porous (nano- and micropores on both surfaces) NGC membranes allow the penetration of oxygen and nutrients but prevent the transit of inflammatory cells. Neurotrophic factors and other growth factors released from activated PRP promote the axonal growth in the NGC. **d** PRP in NGCs stimulated the regeneration of Schwann cells and axons by releasing neurotrophic factors. **e** Interposition of an NGCs in our rabbit model of RLN injury. (Right RLN as control, Lt RLN: two different type of NGCs applied). **f** Photo of a left side RLN (arrow). **g** Photo of a PCL/Pluronic F127 NGC tube (arrowhead). NGC nerve guidance conduit, PRP platelet-rich plasma. RLN recurrent laryngeal nerve.

The aim of this study was to evaluate the ability of PRP-loaded PCL/Pluronic F127 membrane NGCs to promote peripheral nerve regeneration (Fig. 1a, b) and to determine whether PRP promotes the proliferation, neurotrophic functions, and migration behaviors of SCs (Fig. 1c, d).

## Results

### Characterization of PRP loaded NGCs

**Fig. 2:**
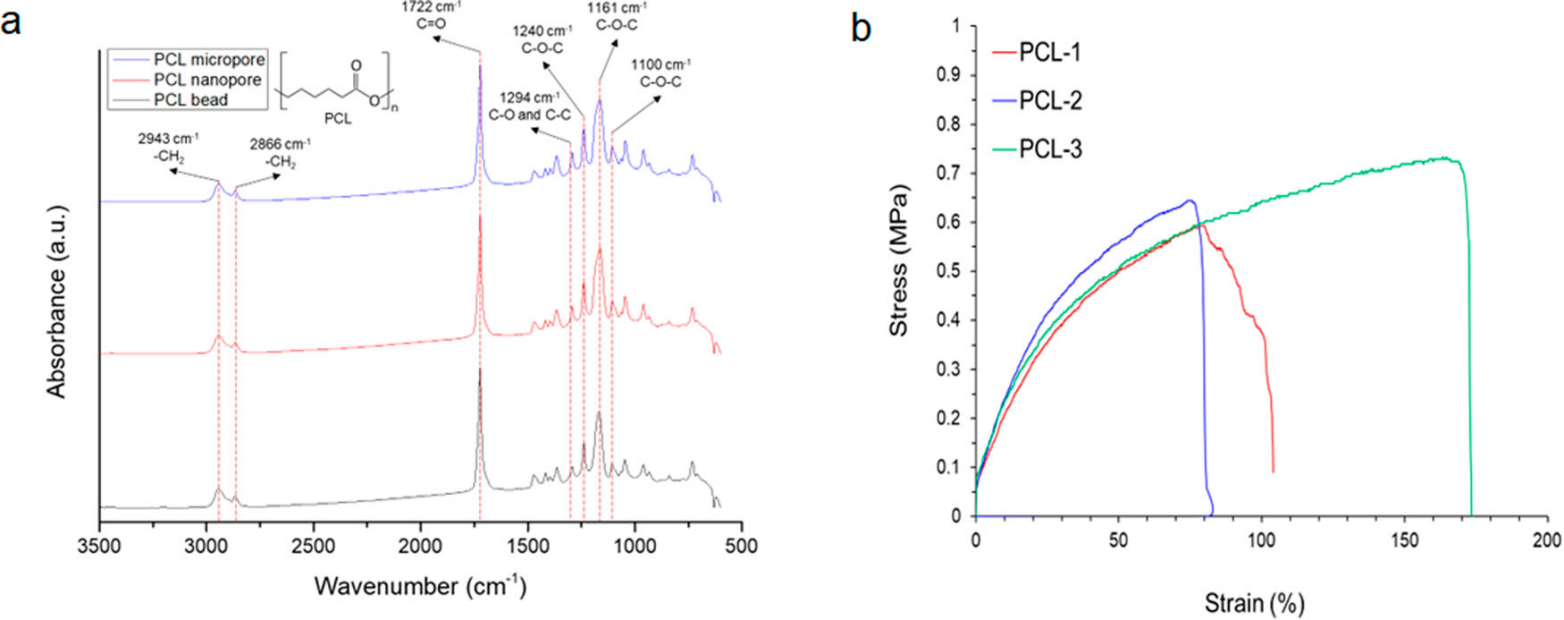
The biomechanical characteristics of the asymmetric PCL/Pluronic F127. **a** FT-IR spectra of PCL bead and PCL membranes. **b** Strain-stress curves of PCL membranes. FT-IR Fourier transform-infrared, PCL polycaprolactone.

We chose a PCL/Pluronic F127 sheet-based NGC system due to its biocompatibility (reviewed in refs. ^16,22,23^) and biomechanical characteristics. First, the biochemical moiety of the PCL membrane was characterized by Fourier transform infrared (FT-IR) vacuum spectrometry. The peaks at 2943 and 2866 cm^−1^ indicate asymmetric −CH_2_ stretching, while the peaks at 1722 cm^−1^ indicated C=O stretching. The peak at 1294 cm^−1^ was related to C–O and C–C stretching. The C–O–C peaks were located at 1240, 1161, and 1100 cm^−1^ (Fig. 2a). All the samples had similar FT-IR spectra before and after asymmetric membrane processing with Pluronic F127, whose chemical moiety is similar to that of PCL. Thus, the FT-IR spectra of the PCL membrane and PCL bead did not differ from each other. From a chemical perspective, the PCL membrane provides very similar functional surface qualities as the pure PCL material. Regarding the mechanical properties of the PCL membranes, Young’s modulus and tensile strength were 2.15 ± 0.98 MPa and 0.66 ± 0.06 MPa, respectively, which is similar to previously reported values^23^. With this stretchable softness and relatively uniform strength, the membrane can provide reliable and relevant matrix environments for nerve or muscular tissues even under vibration, rotation, twisting, and stretching (Table 1).

**Table 1:** Mechanical properties of PCL membranes.

Mechanical Properties	Young’s modulus (MPa)	Strain-to-failure (%)	Tensile strength (MPa)
PCL membrane	2.15 ± 0.98	119.55 ± 39.38	0.66 ± 0.06

**Fig. 3:**
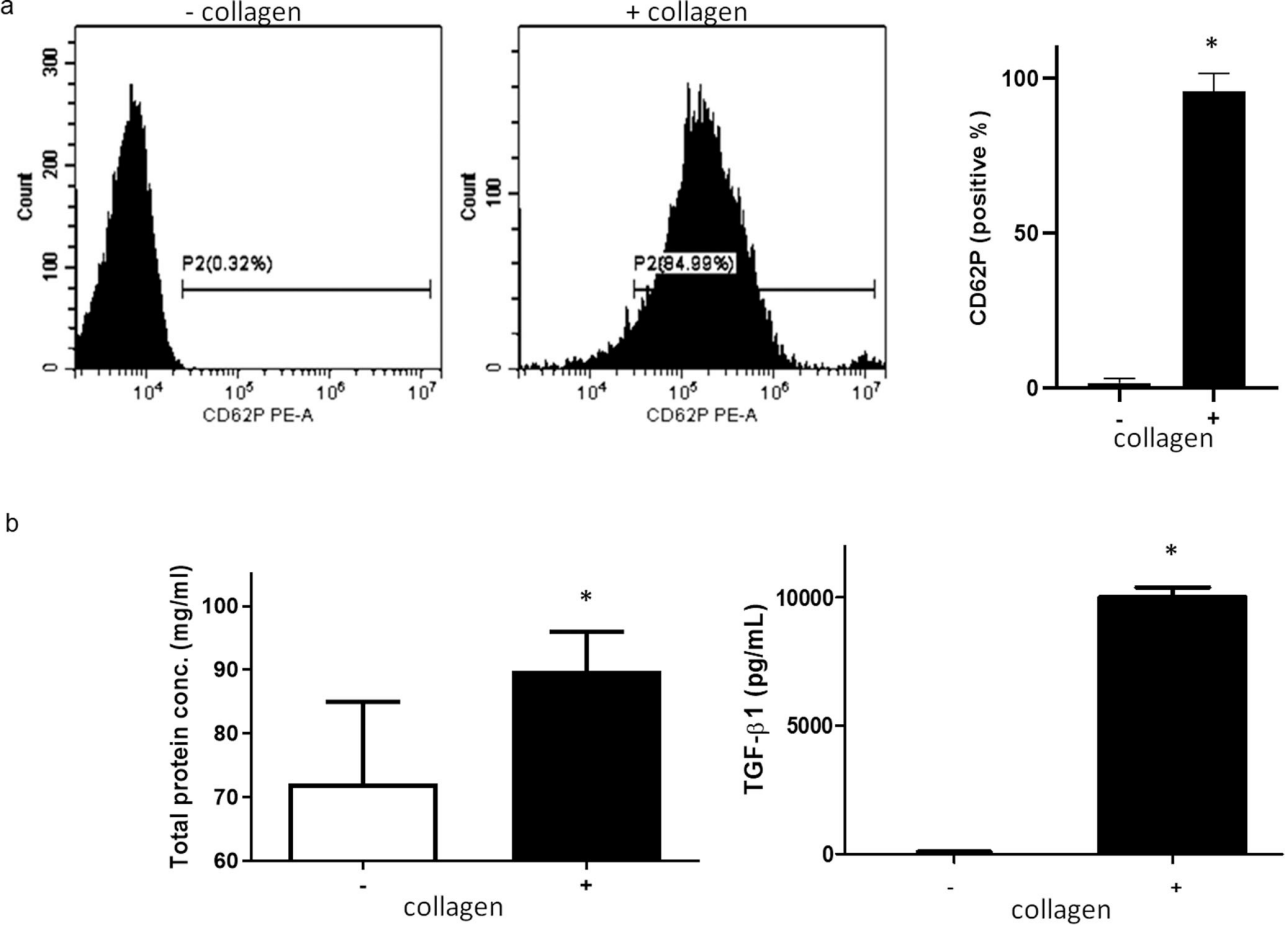
Activation of PRP. **a** Detection of platelet activation marker CD62 by FACS. **b** Left: Total protein levels in activated PRP was significantly higher than in resting state. Right: By ELISA assay, the level of TGF-β in activated PRP was significantly higher than resting state (*, *P* < 0.0001). PRP, platelet-rich plasma, CD62P cluster of differentiation 62, FACS fluorescence-activated cell sorting, RT PCR reverse transcription polymerase chain reaction, TGF-β transforming growth factor beta.

The validation of PRP gel activation was performed after adding collagen and platelet activation marker, CD62P expression was upregulated in activated PRP as determined by flow cytometry in the collagen-containing gel compared to the collagen-negative sample (Fig. 3a, *P* < 0.0001). Analysis of GF concentrations in activated PRP gel samples revealed that TGF-β1 levels were significantly higher than those in the resting state (Fig. 3b, *P* < 0.0001).

### In vitro effect of PRP on human SCs

**Fig. 4:**
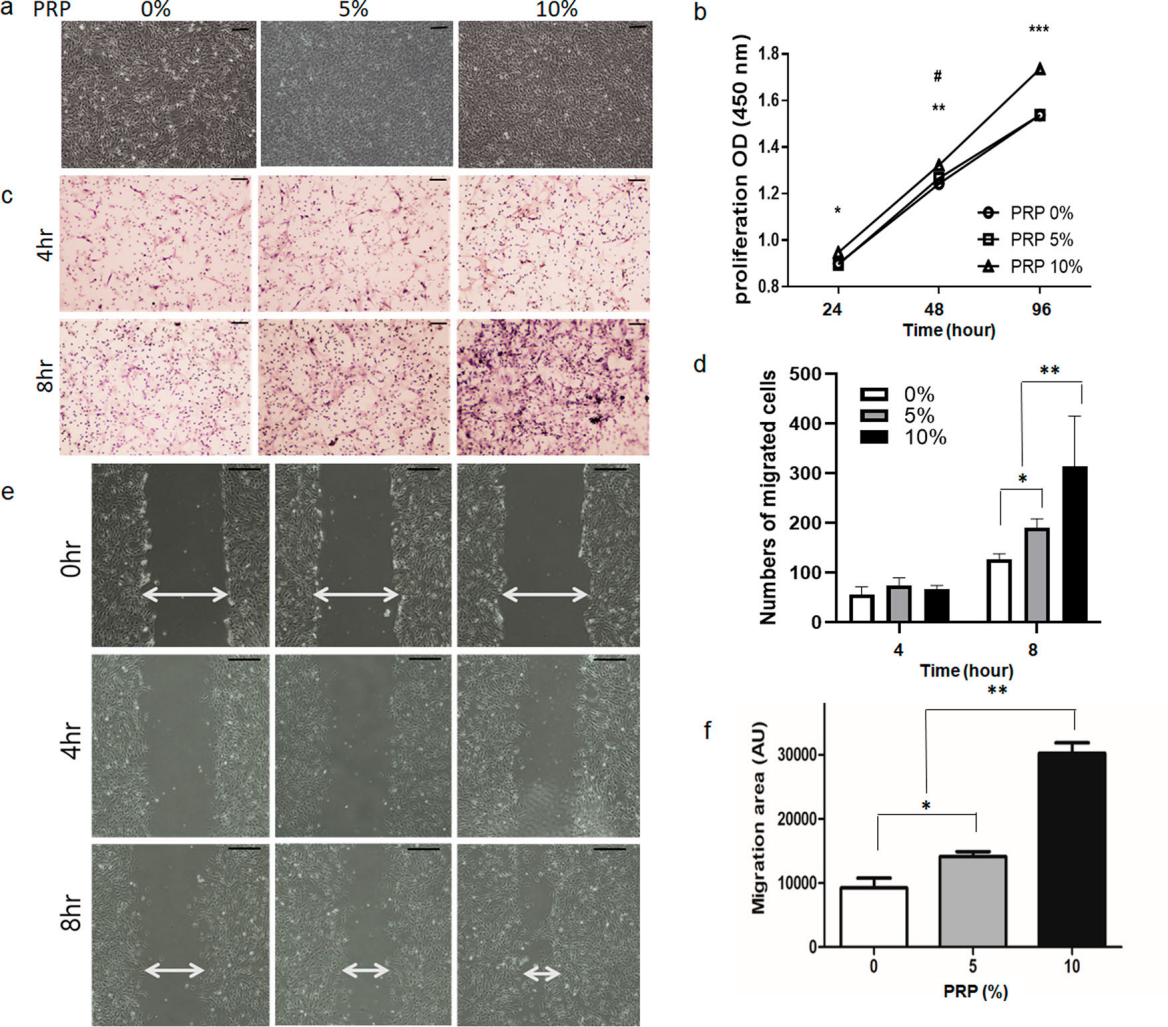
Analysis of the proliferation and migration of human SCs. **a** human SCs exhibited a spindle-shaped and bipolar or occasionally multipolar cell morphology. As the concentration of PRP was increased, the proliferation of human SCs increased. (scale bar, 100 μm). **b** The number of human SCs were increased significantly and dose-dependently by PRP. After treatment for 96 hours, 10% PRP increased SC cell count significantly more than in non-treated controls. (#, compared between 0% and 5%, #*P* < 0.05; *compared between 0% and 10%, *P < 0.05, **P < 0.005, **P < 0.0001). **c** Representative light photomicrographs of migrated human SCs induced by 0%, 5%, and 10% PRP after incubation for 4 or 8 h by the transwell assay (scale bar, 100 μm). **d** Human SCs treated with 5% PRP for 8 h had significantly greater proliferation rates than non-treated controls (*P < 0.05) The number of migrated cells in 10% PRP is higher than in both non-treated cells and 5% PRP treated cells. (**P < 0.0001, respectively). **e** PRP significantly and dose-dependently increased SCs migration rate as compared with non-treated SCs. **f** The migration rate of cells treated with 10% PRP was 4.25-fold greater than that of non-treated controls (*P* < 0.05). SCs Schwann cells, PRP platelet-rich plasma.

To explore the in vivo effect of PRP on cell proliferation, we first investigated its effects on human SC metabolism at various concentrations (0%, 5%, and 10%). At baseline, cells had a typical spindle shape alignment and a bipolar or occasionally multipolar morphology (Fig. 4a). The proliferation of SCs was evaluated using various concentrations of PRP for various times by quantitative analysis (Fig. 4b). On day 1, there was a significant difference between the 0% PRP and 10% PRP groups (*P* = 0.01). However, on day 2, SCs treated with 5% PRP also showed greater cell proliferation than the control (*P* = 0.04). On day 3, SCs with 5% PRP did not demonstrate a significant difference compared to the control (*P* > 0.05), but SCs cultured with 10% PRP showed higher proliferation than those cultured with 0% PRP (*P* = 0.0001). We also compared the effect of different concentrations of PRP on cell migration using a transwell assay. Human SCs treated with PRP at 5% and 10% demonstrated significantly faster migration rates than those without PRP at 8 h in a dose-dependent manner (*P* < 0.05, *P* < 0.0001, respectively) (Fig. 4c, d). Migration was assessed using a scratch wound healing assay. Cells treated with 10% human SCs had significantly higher migration rates than non-treated cells, and the migration rate of cells in the 10% PRP group was 4.25-fold greater than that of non-treated controls (*P* < 0.05) (Fig. 4e, f).

**Fig. 5:**
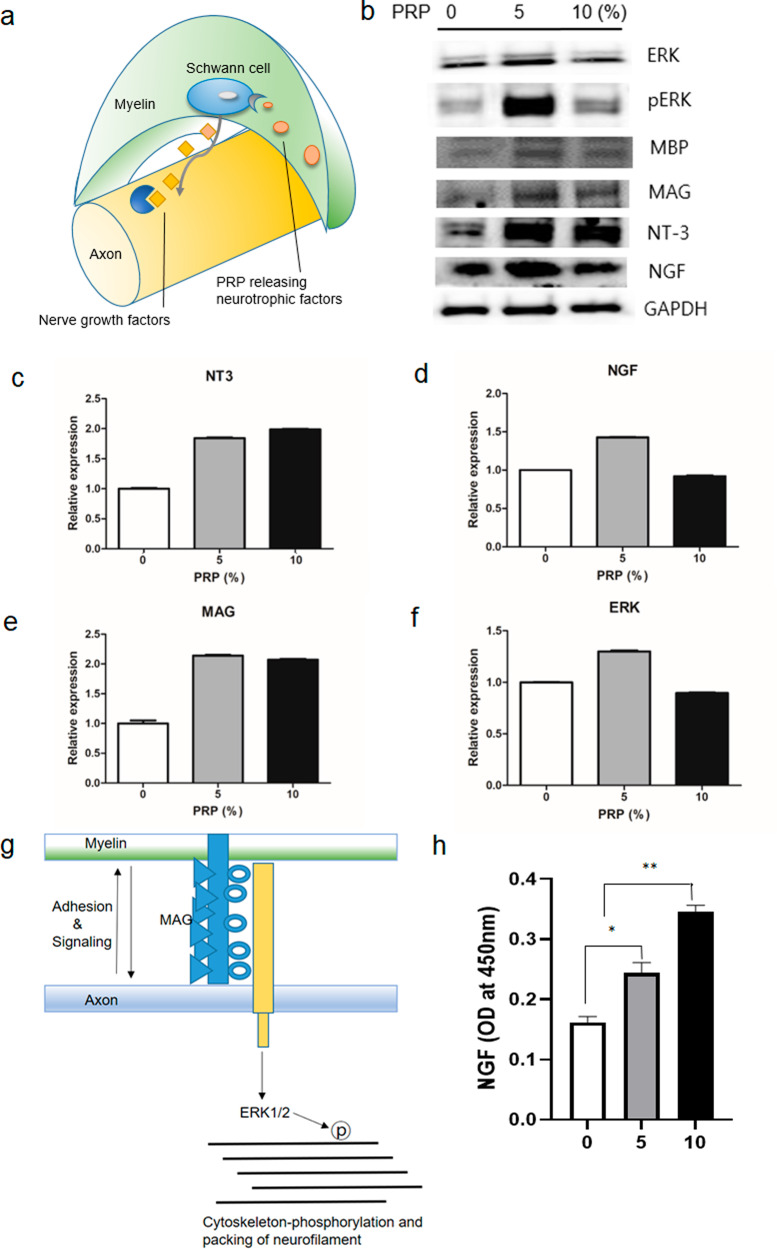
In vitro neurotrophic factor secretion behavior after PRP treatment on human SCs. **a** Schematic illustration of nerve. An axon surrounded by a myelin sheath and Schwann cells, which are located in myelin and release neurotrophic factors. **b** Western blot analysis of neural regeneration-related proteins in human SCs which were treated with different concentrations of PRP. All blots derive from the same experiment and were processed in parallel. **c**, **d** Quantification of neurotrophic factors including NT3 and NGF. As the concentration of PRP increased the expressions of neurotrophic factors increased more so than that observed in non-treated controls. **e**, **f** MAG expression was increased dose-dependently by PRP. ERK (a MAG-related axon cytoskeletal protein) expression was also increased by PRP. **g** Schematic illustration of connection between axon and myelin. **h** The expression of NGF in human SCs by ELISA; human SCs were observed to express NGF in a PRP dose-dependent manner. SC Schwann cell, PRP platelet-rich plasma, NGF nerve growth factor, NT-3 neurotrophin-3, MAG myelin-associated glycoprotein, MBP myelin basic protein, ERK extracellular signal-regulated kinase protein.

As shown in the schematic illustration in Fig. 5a, the myelins, which encircle and protect the axon structures, have multiple SCs, and promote axonal growth by secreting nerve growth factors. The expression of proliferation-related proteins in human SC proteins treated with different PRP concentrations at 0, 5, and 10%. After treatment with PRP at concentrations of 0%, 5%, and 10%, the expression of neurotrophic factors, including NGF and NT-3, was higher compared with that of non-treated controls (Fig. 5b). In particular, NT-3 expression was significantly higher in the 5% PRP-treated cells than in the untreated cells (*P* < 0.05, Fig. 5c). In addition, NGF expression was higher in the 5% PRP-treated cells than in the untreated controls (*P* < 0.05, Fig. 5d). The expression of MAG, which is an adhesion molecule between myelin and axon, acts for signaling to axon cytoskeleton-phosphorylation, and MBP (myelin basic protein) were increased by PRP (*P* < 0.05, Fig. 5b, e). The expression of ERK (a MAG-related axon cytoskeleton protein) in the 5% PRP-treated cells was higher than that in non-treated cells (Fig. 5f). The expression of NT-3, MBP, and MAG was increased in the 5% and 10% PRP treatment groups compared to that of the 0% PRP treatment and did not show dose dependency. This tendency supports the schematic illustration shown in Fig. 5g. Human NGF, which originated from human SCs, increased significantly with the concentration of rat PRP (*P* < 0.05, Fig. 5h). The expression of NGF in Schwann cells was increased only in 5% PRP compared to 0% PRP, whereas only secreted NGF levels from Schwann cells were dose-dependently increased in 5% and 10% PRP compared to non-treated controls.

### In vivo effect of PRP-loaded NGCs

**Fig. 6:**
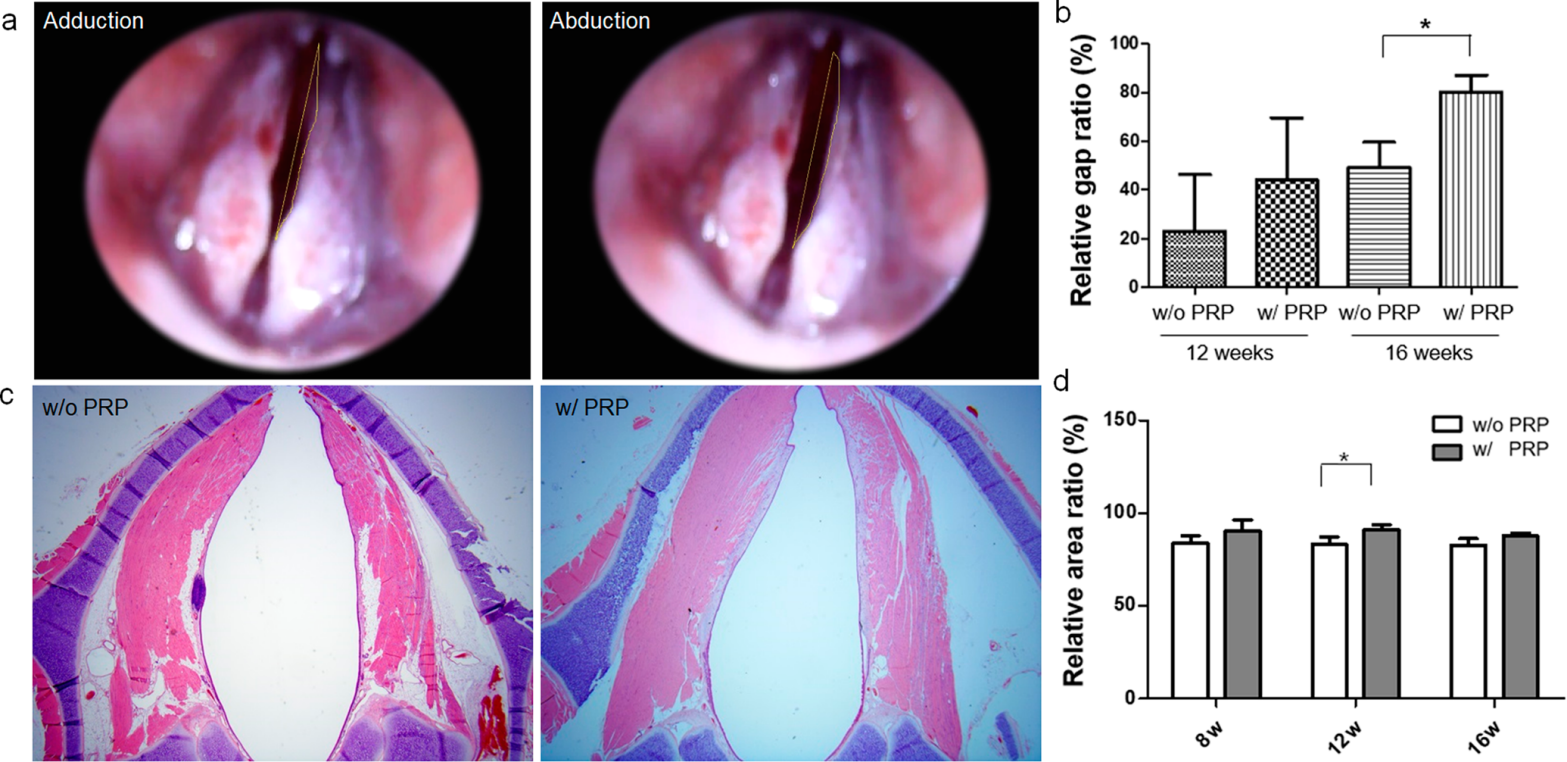
Endoscopic examinations of vocal cord movement and histologic analysis of vocalis muscle. **a** Captured images of the adducted position after nerve stimulation and of the abducted position. A video clip (Video S2) of both vocal cord movement after electrical stimulation is available in the Supplementary Materials. **b** Relative gap ratios between vocal cords in adduction and abduction were significantly greater in the PRP-loaded NGC group (80.23 ± 6.91%) than in the non-loaded group (48.99 ± 10.71%) (*P* = 0.018). **c** Axial section of vocalis muscle: in the PRP group, muscle thicknesses were similar on left and right sides, but in the non-loaded group, left-side muscles were atrophied. **d** The PRP group had significant higher vocalis muscle area ratios than the non-loaded group at 12 weeks post-implantation (*P* = 0.033). NGC nerve guidance conduit, PRP platelet-rich plasma.

Endoscopic images of vocal cord movement were obtained at 8, 12, and 16 weeks after NGC implantation. We recorded the video clip (Video S1) immediately after segmental resection; however, there was no visually marked volitional movement. Recovery of vocal cord movement was not observed at eight weeks post-implantation in the PRP-loaded NGC or non-loaded NGC groups. At 12 weeks post-implantation, restoration of vocal cord movement was observed in 1 of 3 (33.3%) rabbits in the non-loaded NGC group and 2 of 4 (50%) in the PRP-loaded NGC group, and at 16 weeks post-implantation, was observed in 2 of 4 (50%) rabbits in the non-loaded NGC group and in 4 of 5 (80%) rabbits in the PRP-loaded NGC group. Relative gap ratios between vocal cord adduction and abduction (Fig. 6a) showed the gap ratio was significantly higher in the PRP group (80.23 ± 6.91%) than in the non-loaded NGC group (48.99 ± 10.71%) (*P* = 0.018) (Fig. 6b).

In addition, we compared muscle thickness as determined in the vocalis muscle cross-sections (Fig. 6c). At 16 weeks post-implantation, axial sections of the right and left side vocalis muscles were similar in the PRP-loaded NGC group, but the left-side vocalis muscles were atrophied in the non-loaded NGC group. Vocalis muscle area ratios were also found to be significantly higher in the PRP-loaded NGC group (89.34 ± 6.23%) than in the non-loaded NGC group (83.20 ± 6.31%) (*P* = 0.033) at 12 weeks postoperatively (Fig. 6d). At week 16, PRP loaded NGC vs non-loaded NGC group the noted vocalis muscle area ratios value as 87.58 ± 0.97% vs 82.56 ± 6.96%, respectively.

**Fig. 7:**
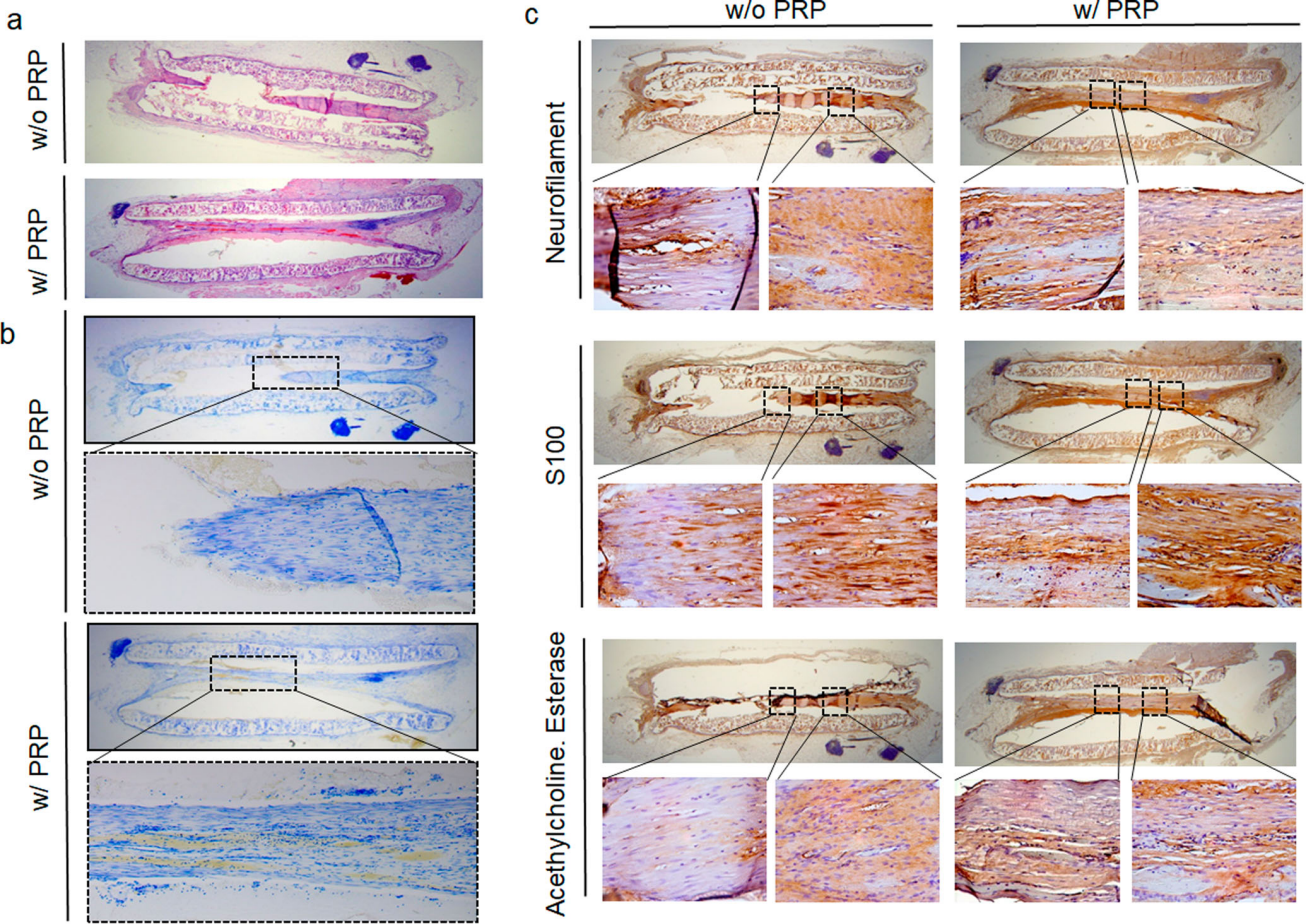
Histologic evaluation and immunohistochemistry analysis of regenerated RLNs. **a, b** H&E and toluidine blue staining demonstrated the longitudinal sections along with RLN at 16 weeks post-implantation showing gradual nerve regeneration within NGCs. Nerve budding from each side connected at 16 weeks in the PRP-loaded group. **c** Immunohistochemistry for NF, S100, and AchE at 16 weeks post-implantation showed strong positivity in the PRP group but not in the non-loaded group. H&E hematoxylin and eosin staining, RLN recurrent laryngeal nerve, NGC nerve guidance conduit, PRP platelet-rich plasma, NF neurofilament; S100, S100 protein, AchE acetylcholinesterase.

Longitudinal sections taken along the RLN at 8, 12, and 16 weeks post-implantation showed gradual nerve growth within the NGCs. At 8 weeks post-implantation, short structural segments originating from nerve stumps were observed in the PRP group but not in the non-loaded PRP NGC group, and a long connected nerve attached to both stumps was observed at 16 weeks in the PRP-loaded NGC group (Fig. 7a). Toluidine blue staining also showed a connection between both nerve endings at 16 weeks in the longitudinal section of the PRP-loaded NGC tube (Fig. 7b). Immunohistochemistry was performed to confirm whether the budding structure was a neural tissue. As shown in Fig. 7c, NF, S100, and ACh esterase were strongly expressed in connected nerve tissue in the PRP-loaded NGC group but were not weakly detected in the non-loaded NGC group at 16 weeks post-implantation.

**Fig. 8:**
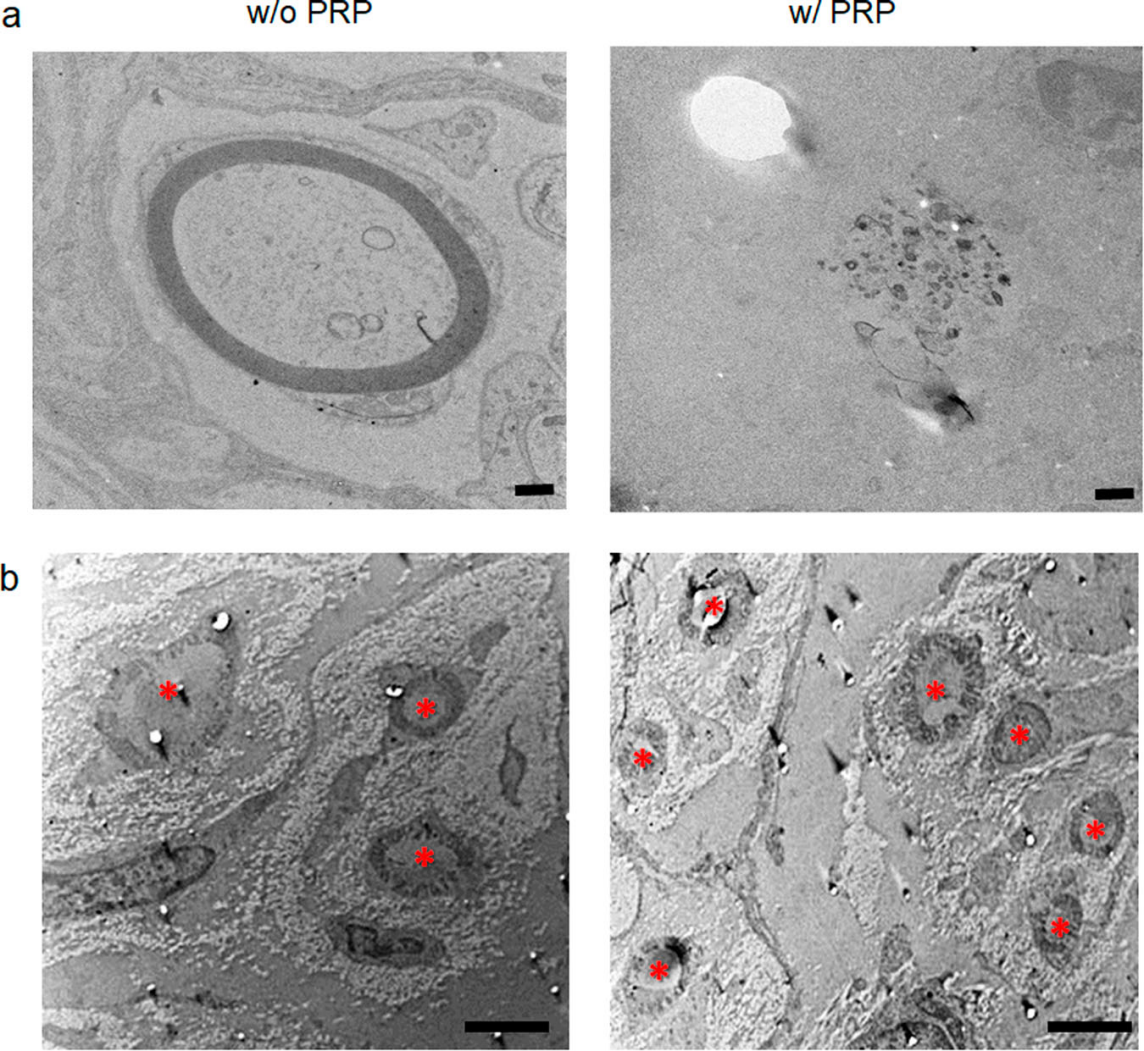
Transmission electron microscopy (TEM) findings at 8 and 16 weeks post-implantation. **a** TEM image at 8 weeks post-implantation (scale bar, 500 nm). **b** TEM images at 16 weeks pot-implantation (scale bar, 2 μm). Asterisks indicates the axons. Myelinated fiber formation was more abundant in the PRP group than in the non-loaded group. Axon fibers in the PRP group at 16 weeks were denser and better organized with Schwann cells than at 8 weeks. NGC nerve guidance conduit, PRP platelet-rich plasma.

TEM images of the mid-region of regenerated nerves in the PRP-loaded NGC group at 16 weeks (Fig. 8b) showed that myelinated fibers were more abundant compared with the non-loaded NGC group and that axon fibers were denser with well-organized myelin and SCs than were observed at 8 weeks post-implantation (Fig. 8a).

## Discussion

Nerves are electrophysiologic and direction-orientated tissues, suggesting that a guiding conduit containing a biomimetic microenvironment might be needed to enhance and direct peripheral nerve regeneration^24,25^. Asymmetrically porous PCL/Pluronic F127 NGCs provide an excellent environment that stimulates nerve growth and axoglial signaling^16^, and its biodegradability improves cell/tissue compatibility^22,23^. Furthermore, in the present study, biochemical and mechanical analyses showed that the PCL properties are very uniform, even though the PCL membrane itself has some degree of non-uniformity and asymmetry. On the other hand, the elongation was also observed up to a hundred times that of the soft elastomer. Therefore, it seems to provide a stable matrix environment that can accommodate all the mechanical events in the tissue.

The optimal PRP concentrations for human SC proliferation and migration are arguable. Zheng et al. performed experiments on the optimal dose of PRP on the proliferation and migration of SCs over 7 days^26^. In their study, on day 1, there were no significant differences between the doses. However, on day 3, increased proliferation was detected in a PRP dose-dependent manner, with the exception of 40%. These data are consistent with our finding that 10% PRP most strongly promoted the proliferation and migration of SCs on day 3. In our study, 5% PRP showed greater proliferation than the control on day 2. However, on day 3, there was no significant difference compared to the control, indicating that certain concentrations of PRP beyond a certain time could decrease the activity of human SCs. PRP is a pool of growth factors and other bioactive mediators, most of which are trapped in a fibrin matrix and bound to fibrin heparin sulfate–binding domains. The fibrin matrix considerably influences the strength, growth factor-trapping, and release potential of PRP. Finally, we concluded that 10% PRP strongly promoted the proliferation and migration of SCs. On the other hand, Zheng et al. demonstrated that 20% PRP significantly stimulated SC proliferation and migration compared to untreated cells^26^. However, they performed an additional centrifugation step after the standard double-spin method, followed by collection of the supernatant for use as PRP, which contained lower concentrations of cytokines. We prepared PRP using a standard method widely used in clinical settings. The different procedures for PRP preparation may explain this difference.

On the other hand, PRP contains biomolecules and GFs that participate in the biological processes involved in nerve regeneration. In this study, we found that PRP-loaded NGCs regenerated rabbit RLNs more efficiently than hollow NGCs. Although a few reports have been published on the usefulness of PRP and NGCs for nerve regeneration, this is the first report to demonstrate the efficacy of PRP-filled NGCs.

Alpha granules of platelets contain GFs with mitogenic and chemotactic characteristics, such as PDGF, TGF-β, IGF-1, FGF, and VEGF. GFs generated by SCs, such as nerve growth factor, brain-derived neurotrophic factor, ciliary neurotrophic factor, and glial cell line-derived neurotrophic factor, are known to be involved in nerve recovery after injury^27–29^.

SCs have been shown to play crucial roles in peripheral nerve regeneration. Following peripheral nerve injury, SCs proliferate, form a Büngner belt, and together with macrophages, devour the debris of denatured axons and myelin. At the same time, SCs express and secrete neurotrophic factors including NGF, neurotrophin-3 (NT-3), neurotrophin-4/5 (NT-4/5), and brain-derived neurotrophic factor (BDNF), which play neuron-protective and axon-inductive roles^30,31^. Qin et al. demonstrated that concentrated GFs increase SC proliferation and neurotrophic factor secretion and promote functional nerve recovery in vivo^32^. In this study, we investigated the NGF-secreting effect of PRP on human SCs in the absence of NGF secreted from rat PRP. Therefore, we used an ELISA kit for detecting human NGF (secreted from human SCs) and for ruling out the secretion of NGF from rat PRP. The expression of NGF in Schwann cells was increased only in 5% PRP compared to 0% PRP, whereas only secreted NGF levels from Schwann cells were dose-dependently increased in 5% and 10% PRP compared to non-treated controls. This suggests that PRP might stimulate the NGF secretion by stimulating SCs, and the increased dose of PRP can promote SC proliferation.

After nerve injury, the migration of SCs is the main mechanism that supports nerve regeneration^33,34^. The significant effect of PRP on SC migration observed in the present study suggests that neurotrophic factors such as NT-3 and NGF in PRP play essential roles in the promotion of SC migration.

MAG is a molecule expressed by myelinating cells at the myelin/axon interface which helps to establish the normal formation and maintenance of myelinated axons^35–38^. MAG is a ligand that binds to an axonal receptor, and this binding activates cyclin-dependent kinase 5 (cdk5) and extracellular signal-regulated kinases 1 and 2 (ERK1/2) signaling and increases the expression of phosphorylated neurofilaments, which in turn, increase axonal maintenance and survival^37^. Our results showed that PRP enhanced the expression of MAG and MBP, which are major proteins of the myelin sheath^36–38^. Yao et al. demonstrated that insulin-like growth factor I (IGF-I) treatment upregulates the gene expression of MBP in an experimental model of encephalomyelitis^39^ and that in oligodendrocytes, IGF-I affects myelin protein synthesis and myelin regeneration^39–41^. IGF-I acts as a neurotrophic factor that promotes peripheral nerve growth and inhibits neuronal and glial apoptosis^42,43^. We found that MBP, which was induced by PRP, could protect neurons from apoptosis through the IGF-I in PRP^40,41,44^ and work in a synergetic fashion with all involved other growth factors in PRP, such as platelet-derived growth factor, vascular endothelial growth factor, and epithelial growth factor for nerve regeneration.

As shown in Fig. 5g, MAG and MBP are the main adhesion and signaling proteins of SCs and are also required for cytoskeleton construction and the packing of axons in neurofilaments^40,41^. The present study suggests that PRP promotes axonal growth by stimulating SCs.

In a previous study, PCL/F127 NGC facilitated rapid bridging of a 10-mm nerve gap and promoted neural tissue growth at 8 weeks post-implantation, which was more than what was observed in the silicone tube group^2^. In the present study, neural regeneration was greater at 16 weeks than at 8 weeks post-implantation in the PRP group, and axial TEM images revealed the presence of dense axon bundles, suggesting that nerves grew well in the GF-abundant environment provided by PRP. The significance of the role played by GFs within PRP was also highlighted in a rat brain-spinal cord co-culture system, in which PRP supernatant increased in axon sizes and numbers^27^.

A multicellular and pleiotropic molecular response is activated after peripheral nerve injury. This response interplays with, and is mainly modulated by, injured axons, myelin breakdown products, soluble factors, and hypoxia^45–48^ and results in the regrowth and guiding of axons at a rate of about 1 mm per day and in their reconnection with target organs^49–51^. SCs exhibit a striking plastic response to nerve damage and are the first entities to be exposed and detect nerve damage. In a context- and time-dependent manner, transdifferentiated SCs perform a variety of cellular repair tasks that range from phagocytosing myelin debris to secreting neurotrophic and neurotropic factors (laminin). Furthermore, proliferating and migrating SCs form cords and Büngner bands in the proximal and distal nerve segments, respectively.

Poor functional recovery after peripheral nerve injury has been attributed to the misdirection of regenerating axons to reinnervate functionally inappropriate muscles. The RLN is a branch of the vagus nerve (cranial nerve X) that supplies all the intrinsic muscles of the larynx, including the vocalis, posterior cricoarytenoid, lateral cricoarytenoid, and interarytenoid muscles. Even though we could not evaluate all target muscles, vocal cord movement after evoked stimulation at the proximal part was demonstrated. Several studies^52,53^ using sciatic nerves showed that the NGC improved the misdirection of axons compared to nerve autografting or direct anastomosis. After nerve injury, the neuromuscular junction (NMJ) results in remodeling, which increases the fragment number, while individual muscle fiber cell membranes spontaneously create fibrillation potential. At the same time, the target muscles gradually atrophy, and muscle wet weight and muscle fiber diameter gradually decrease. Ma et al. showed that even if the SC number normalizes and targets muscle atrophy severely, muscle wet weight is reduced to 15%^54^. In this study, a slightly smaller vocalis muscle ratio at week 16 compared to that at week 12 represents severe muscle atrophy during nerve regeneration.

The present study has several limitations that warrant consideration. First, the gap created in the RLNs was 10-mm, but the maximum gap and appropriate timing of implantation after injury were not evaluated. This NGC model might be typically necessary in cancer resection settings and would be less applicable in the setting of nerve transection. Second, we did not compare the efficacy of neural regeneration between the end-to-end epineural suture group and the PRP-loaded NGC group. However, Ikumi et al. compared autologous grafting using an epineural suture and an autologous graft coated with activated PRP and found that local PRP administration increased regenerative axon diameters and axon numbers in the distal portion^55^. Third, the proliferation of SCs is important in nerve regeneration, but we did not perform experiments with a neural cell model. However, the effects of PRP on model neural cells have been demonstrated in previous studies. Baklaushev et al. showed that a PRP-derived hydrogel dramatically stimulated proliferation and neuronal differentiation of directly reprogrammed human neural precursor cells due to the complex action of the PRP components and creation of a 3D biomimetic environment^56^. In addition, Li et al. demonstrated that human adipose stem cells (hASCs) treated with PRP displayed higher levels of neuron-specific enolase and membrane-associated protein-2 compared to the control group. These results indicate that PRP is capable of promoting cell proliferation and neurogenic differentiation of hASCs in vitro^57^. Finally, we did not compare gelatin-filled NGCs (vehicle control) and PRP-filled NGCs. Further studies are required to address these issues.

In summary, the present study demonstrates the therapeutic potential of an NGC system loaded with PRP for nerve regeneration both in vitro and in vivo. In terms of RLN functional regeneration, PRP-loaded NGCs produced better results than non-loaded NGCs. The PRP group exhibited less vocalis muscle atrophy, faster nerve growth, and greater well-organized axon density. It appears that PRP in NGCs improves neural cell proliferation and regeneration in a GF-rich environment. We found that GF**s** in PRP increased SC proliferation and that PRP promoted SCs to secrete neurotrophic factors in vitro, and promoted functional recovery after peripheral nerve injury in vivo. Our findings show that PRP-loaded NGCs provide a favorable environment for neural regeneration and suggest that they offer a possible therapeutic strategy for promoting RLN recovery.

## Methods

### Preparation and quantification of PRP

Rat blood was drawn from the heart using a disposable syringe containing 2.2% sodium citrate (9:1, v/v). PRP was prepared by centrifugation of citrated blood at 200 g for 10 min at room temperature (22 °C), and platelet-poor plasma (PPP) was obtained from the residue by centrifugation at 1500 × *g* for 20 min. Platelet number was adjusted to 4-4.5–10^8^/mL by mixing PRP and PPP, and the adjusted PRP preparation was used for the following experiments. The adjusted PRP was incubated at 37 °C with continuous stirring at 1200 rpm. PRP was equilibrated at 37 °C for 3 min before the initiation of the experiment. To activate platelets, collagen (10 µg/mL) was added, and the reaction was stopped at 30 min by cooling at 4 °C. The expression of CD62P (platelet activation marker) was measured using a flow cytometer (Beckman Coulter, USA). Total protein concentration was measured by bicinchoninic acid (BCA) protein assay (Thermo Fisher Scientific, USA), while transforming growth factor-beta (TGF-β) was quantified using an enzyme-linked immunosorbent assay (R&D system, USA) following the manufacturer’s instructions.

### NGC preparation

PCL/Pluronic F127 sheets were prepared using an immersion precipitation method, while NGCs were produced by rolling, as previously described^16^. Briefly, to make an asymmetrically porous sheet (nano- and micropores on both surfaces), we dissolved the PCL pellets in tetraglycol (10 wt %; Sigma Aldrich, St Louis, MO) at 90 °C; then, we added Pluronic F127 powder (BASF, Ludwigshafen, Germany) to the PCL solution (5 wt %, PCL base). PCL/F127 (50 mm × 50 mm × 0.4 mm) was molded and immersed for 1 h at room temperature. The precipitated PCL/F127 sheet was washed and vacuum-dried. The sheet was rolled into a tube using a 1 mm diameter metal mandrel (inside the tube, nanopore side), and the edge of the sheet was fixed using a tissue adhesive (Histoacryl; B. Braun, Melsungen, Germany). The prepared asymmetrically porous NGCs had an inner diameter of 1 mm and a length of 10 mm. We validated the chemical moiety of the PCL membrane using a Fourier transform-infrared vacuum spectrometer (FT-IR; VERTEX 80 V, Bruker). The mechanical properties of the PCL membranes were measured using a tensile tester (BMSTP-50PPA, BMS Tech., Korea). The specimens were prepared in a dog-bone shape (width: 0.3 mm and length: 0.5 mm).

### Fabrication of PRP alginate mixture in the NGCs

The PRP alginate mixture was fabricated through the internal gelation process, as previously described^58^. Briefly, PRP was mixed with 2% alginate solution made from alginic acid sodium salt derived from brown algae (Sigma, Medium Viscosity). The mixture was then dispensed via a syringe needle (26½ gauge) inside the NGC tube blending in 6% CaCl_2_ (Sigma). The PRP plus alginate mixture was gelled by the diffusion of Ca^2+^ ions into the polymer mixture. After gelation, the beads were incubated in CaCl_2_ solution for 20 min to complete the gelation process.

### Preparation of Schwann cells (SCs)

Human SCs were purchased from ScienCell Research Laboratories (catalog no.1700; Carlsbad, CA) and cultured in Schwann cell medium (SCM, ScienCell Research Laboratories, catalog no.1701, Carlsbad, CA) in poly-L-lysine (2 ug/cm^2^)-coated culture dishes. The medium was changed every three days until the culture was approximately 90% confluent.

### Cell proliferation

Primary SCs were seeded in 96 well plates at 1 × 10^4^ cells per well, and cultured in culture medium with PRP at concentrations of 0%, 5%, or 10%, which were chosen based on previous reports reviewed in refs. ^26,59,60^. Cell proliferation was evaluated using the Cell Counting Kit-8 assay (CCK-8, Dojindo, Japan) on culture days 1 and 3. After adding 10 µL of CCK-8 reagent and incubation at 37 °C for 3 h, formazan absorbance was measured at 450 nm using a well plate reader (Dynex Revelation, Dynex Ltd, UK).

### Cell migration and wound healing assay

Cell migration was assessed using a transwell assay (Corning Inc., Corning, NY) and wound healing assay. For transwell assays, SCs were introduced into top chambers in culture medium and different concentrations (0%, 5%, or 10%) of PRP gel were added to the lower chambers. After 4 and 8 h, non-migrated cells on the upper membrane surfaces were removed using cotton swabs, while migrated SCs on lower surfaces were fixed with methanol and stained with crystal violet solution. Migrated cells were imaged and counted under a microscope (Olympus, Japan). The number of migrated cells was expressed as percentages of migrated non-treated controls.

For the wound healing assay, SCs were grown to confluence in SCM containing 0%, 5%, and 10% PRP gel. Confluent layers were then ‘wounded’ using a sterile 100 µL pipette tip, and 4 and 8 h later, migration rates into wounded areas were measured using an inverted microscope (Olympus, Japan). Migration areas are expressed as percentages of those of PRP-untreated controls.

### Protein expressions

Western blotting was performed to assess the changes in protein expression in human SCs treated with PRP at 0%, 5%, and 10% concentrations. The primary antibodies used were as follows: myelin-associated glycoprotein (MAG, Santa Cruz Biotechnology, Santa Cruz, CA), myelin basic protein (MBP, Santa Cruz Biotechnology, Santa Cruz, CA), neurotrophin-3 (NT-3, Santa Cruz Biotechnology, Santa Cruz, CA), nerve growth factor (NGF, Santa Cruz Biotechnology, Santa Cruz, CA), and extracellular signal-regulated kinase (ERK; Santa Cruz Biotechnology, Santa Cruz, CA). GAPDH (Santa Cruz Biotechnology, Santa Cruz, CA, USA) was used as an internal control. All blots derive from the same experiment and were processed in parallel. Quantitative analysis was performed using ImageJ software (version 1.49; Wayne Rasband, National Institutes of Health). To rule out the expression of NGF in rat PRP, NGF concentrations in SC-media were determined by ELISA (Abnova, Taipei, Taiwan) according to the manufacturer’s instructions.

### Rabbit recurrent laryngeal nerve (RLN) injury model

This study was approved by the Animal Ethics Committee of Inha University Hospital (INHA 18 0503-560) and animal care and experiments were performed in accordance with established institutional animal ethics committee regulations. Twenty-two female New Zealand white rabbits about 2 kg at 12 weeks of age were randomly assigned to a non-loaded NGC group (*n* = 10) or a PRP-loaded NGC tube group (*n* = 12). The right RLNs of rabbits were preserved as normal controls, but the left RLNs were resected and interposed with an NGC (Fig. 1e). Preoperatively, animals were anesthetized with 5 mg/kg subcutaneous xylazine, and immediately prior to surgery, an intramuscular injection of 15 mg/kg zolazepam was administered. In each case, a cervical vertical incision was used to expose the left RLN, which was then dissected circumferentially (Fig. 1f, g). A 10-mm length was resected. An NGC was interposed between the proximal and distal nerve endings and attached using junction sutures (7-0 Vicryl; Ethicon, Somerville, NJ, USA). A total of 10 rabbits underwent non-loaded NGC tube interposition and 12 rabbits underwent PRP-loaded NGC tube interposition. After implantation, the strap muscles were closed with a 4-0 Vicryl suture, and the skin was closed with 3-0 Ethilon (Ethicon, Somerville, NJ).

### Evaluation of vocal cord movements

Endoscopic laryngeal examinations were performed at 8, 12, and 16 weeks after the NGC implantation. Briefly, a rabbit was anesthetized with 5 mg/kg subcutaneous xylazine and intramuscular injection of 15 mg/kg zolazepam, and the neck wound of an experimental animal was opened in the supine position. First, we found an interposed tube on the left side of the neck. After exposure of the inserted tube on the left side and normal RLN on the right side, we performed laryngoscopic examination by inserting a rigid laryngeal endoscope (Storz, Tuttlingen, Germany). Then, we stimulated a 2-mm site proximal to the resected region using a nerve stimulator (Maplewood; WR Medical Electronics Co., Saint Paul, MN) with an electric current of 1.5 mA. After nerve stimulation, we checked vocal cord movements, captured images in adducted and abducted positions, and calculated mean vocal cord gap ratios (injured left side versus normal right side) in the non-loaded NGC and PRP-loaded NGC groups. Restoration of vocal cord movement was evaluated using a binary scale (yes/no). We also calculated the relative gap ratio (left gap/right gap × 100), and the gap was measured with the triangular areas in the fully abducted and adducted positions using Image J (NIH, Bethesda, MD).

### Histological examination

After the endoscopic vocal cord evaluation, the animals were sacrificed. NGCs, including RLNs and nerve stumps, were harvested to evaluate nerve growth, and larynges were resected to assess the vocalis muscle status. NGC tubes including RLNs and larynges were fixed in 4% formaldehyde, immediately embedded in paraffin, and sectioned (NGCs were sectioned longitudinally, and the vocalis muscles of larynges were sectioned axially). Longitudinal sections of NGCs were stained with hematoxylin and eosin (H&E) and toluidine blue, while vocal process level of the vocalis muscles were stained with H&E. A single-blinded observer measured the cross-sectional areas of the vocalis muscles using ImageJ software by tracing the outlines of the microscopic images. Areas of the left side denervated vocalis muscles were expressed as percentages of those of the normal right side muscles.

### Immunohistochemical analysis

To identify specific neural markers in regenerated tissue inside NGCs, we performed immunohistochemical analyses using AChE (AchE, Abcam, Cambridge, UK), neurofilament (NF, Thermo Fisher Scientific, Waltham, MA, USA), and anti-S100 protein (Merck Millipore, Darmstadt, Germany).

### Transmission electron microscopy

Regenerated nerve structures were examined by transmission electron microscopy (TEM; 80 kV; Zeiss, Oberkochen, Germany) to investigate regenerated nerve structures, especially the transverse section of the NGC at 8, 12, and 16 weeks postoperatively. The number of axons was counted, and the overall structures of myelinated fiber bundles were noted.

### Statistical analysis

The Kruskal Wallis and Mann–Whitney tests were used to determine the significance of the effects of different PRP concentrations on proliferation, migration, wound healing, and the expression of target proteins. The Mann–Whitney test was used to determine the difference in vocal cord movements, axial section areas of the vocalis muscles, and number of nerve endings in the control and PRP groups. The analysis was performed with GraphPad Prism 5.0 (GraphPad Software, Inc., San Diego, CA), and statistical significance was accepted for *p* values < 0.05.

### Reporting summary

Further information on research design is available in the Nature Research Reporting Summary linked to this article.

## Supplementary information

Supplemental materials

Video S1

Video S2

REPORTING SUMMARY

## Data Availability

The data that support the findings of this study are available from the corresponding author upon reasonable request.
