# Supplementary material for: Platelet-rich plasma loaded nerve guidance conduit as implantable biocompatible materials for recurrent laryngeal nerve regeneration
Source: NPJ Regen Med. 2022 Sep 14;7:49. doi: 10.1038/s41536-022-00239-2 (PMC9474804; doi:10.1038/s41536-022-00239-2)
Supplement: Supplementary file 1 — Supplemental materials [file 41536_2022_239_MOESM1_ESM.docx]

**Supplementary Material**

**Platelet-rich plasma loaded nerve guidance conduit as implantable biocompatible materials for recurrent laryngeal nerve regeneration**

Ji Won Kim, Jeong Mi Kim, Mi Eun Choi, Eun Jeong Jeon, Jin-Mi Park, Young-Mo Kim, Seung-Ho Choi, Taesik Eom, Bong Sup Shim, and Jeong-Seok Choi

**Supporting figures and videos**


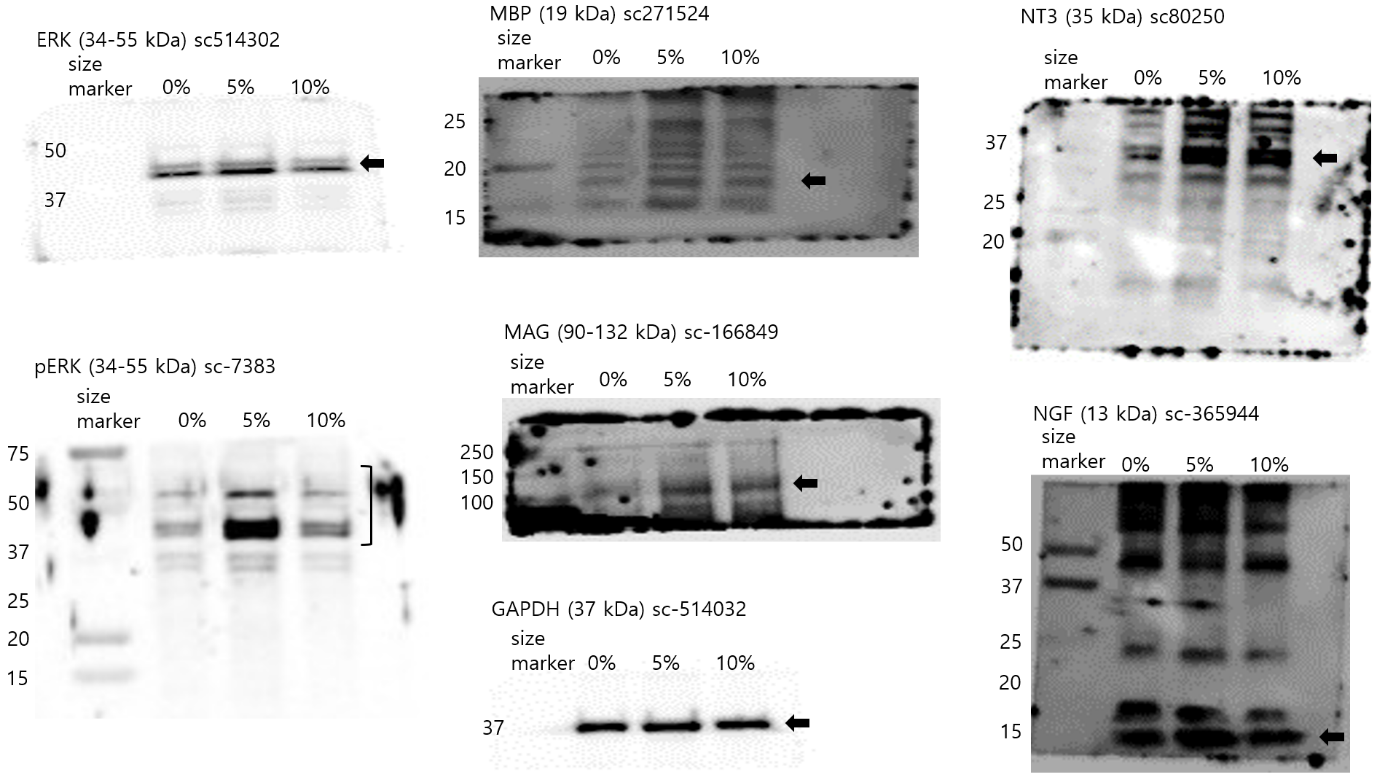


**Supplementary Figure 1.** Uncropped blots relate to figures in this study. Arrows indicate the representative bands used in this main text.

**Supplementary Video 1**. A video clip of typical vocal cord movement when normal breathing at time zero- immediately following resection of the RLN.

**Supplementary Video 2**. A video clip of both vocal cord movement after electrical stimulation in relation to Figure 6.
