# Supplementary material for: Platelet-rich plasma loaded nerve guidance conduit as implantable biocompatible materials for recurrent laryngeal nerve regeneration
Source: NPJ Regen Med. 2022 Sep 14;7:49. doi: 10.1038/s41536-022-00239-2 (PMC9474804; doi:10.1038/s41536-022-00239-2)
Supplement: Supplementary file 4 — REPORTING SUMMARY [file 41536_2022_239_MOESM4_ESM.pdf]

nature portfolio

Corresponding author(s): Professor Jeong-Seok Choi

Last updated by author(s): Jul 23, 2021

Reporting Summary

Nature Portfolio wishes to improve the reproducibility of the work that we publish. This form provides structure for consistency and transparency in reporting. For further information on Nature Portfolio policies, see our Editorial Policies and the Editorial Policy Checklist.

Statistics

For all statistical analyses, confirm that the following items are present in the figure legend, table legend, main text, or Methods section.

n/a	Confirmed	
☐	☒	The exact sample size (n) for each experimental group/condition, given as a discrete number and unit of measurement
☐	☒	A statement on whether measurements were taken from distinct samples or whether the same sample was measured repeatedly
☐	☒	The statistical test(s) used AND whether they are one- or two-sided<br>*Only common tests should be described solely by name; describe more complex techniques in the Methods section.*
☐	☒	A description of all covariates tested
☐	☒	A description of any assumptions or corrections, such as tests of normality and adjustment for multiple comparisons
☐	☒	A full description of the statistical parameters including central tendency (e.g. means) or other basic estimates (e.g. regression coefficient) AND variation (e.g. standard deviation) or associated estimates of uncertainty (e.g. confidence intervals)
☐	☒	For null hypothesis testing, the test statistic (e.g. F, t, r) with confidence intervals, effect sizes, degrees of freedom and P value noted<br>*Give P values as exact values whenever suitable.*
☒	☐	For Bayesian analysis, information on the choice of priors and Markov chain Monte Carlo settings
☒	☐	For hierarchical and complex designs, identification of the appropriate level for tests and full reporting of outcomes
☒	☐	Estimates of effect sizes (e.g. Cohen's d, Pearson's r), indicating how they were calculated

Our web collection on statistics for biologists contains articles on many of the points above.

Software and code

Policy information about availability of computer code

Data collection	We did not use open source data in this study
Data analysis	The analysis was performed with GraphPad Prism 5.0 (GraphPad Software, Inc., San Diego, CA).

For manuscripts utilizing custom algorithms or software that are central to the research but not yet described in published literature, software must be made available to editors and reviewers. We strongly encourage code deposition in a community repository (e.g. GitHub). See the Nature Portfolio guidelines for submitting code & software for further information.

Data

Policy information about availability of data

All manuscripts must include a data availability statement. This statement should provide the following information, where applicable:

- Accession codes, unique identifiers, or web links for publicly available datasets
- A description of any restrictions on data availability
- For clinical datasets or third party data, please ensure that the statement adheres to our policy

Accession codes, unique identifiers, or web links for publicly available datasets

Field-specific reporting

Please select the one below that is the best fit for your research. If you are not sure, read the appropriate sections before making your selection.

☒ Life sciences ☐ Behavioural & social sciences ☐ Ecological, evolutionary & environmental sciences

For a reference copy of the document with all sections, see nature.com/documents/nr-reporting-summary-flat.pdf

Life sciences study design

All studies must disclose on these points even when the disclosure is negative.

Sample size	To determine the sample sample size, we consider the the idea about significant difference between the groups should be taken preferably from previously published studies. For sample size calculation, it is important to have an idea about statistical test, such as Kruskal Wallis and Mann–Whitney tests. Furthermore, we expected 10% attrition then decided the final sample size.
Data exclusions	No data were excluded from the analyses.
Replication	All attempts at replication were successful.
Randomization	All animals we used were allocated into experimental groups. However, the side using the experimental materials was compared with the normal one in one individual. Type of materials used were randomly assigned in the order of the experiment.
Blinding	Investigators were blinded to group allocation during data collection and analysis.

Reporting for specific materials, systems and methods

We require information from authors about some types of materials, experimental systems and methods used in many studies. Here, indicate whether each material, system or method listed is relevant to your study. If you are not sure if a list item applies to your research, read the appropriate section before selecting a response.

Materials & experimental systems

n/a	Involved in the study	
☐	☒	Antibodies
☐	☒	Eukaryotic cell lines
☒	☐	Palaeontology and archaeology
☐	☒	Animals and other organisms
☒	☐	Human research participants
☒	☐	Clinical data
☒	☐	Dual use research of concern

Methods

n/a	Involved in the study	
☒	☐	ChIP-seq
☐	☒	Flow cytometry
☒	☐	MRI-based neuroimaging

Antibodies

Antibodies used	myelin-associated glycoprotein (MAG, Santa Cruz Biotechnology, Santa Cruz, CA),<br>myelin basic protein (MBP, Santa Cruz Biotechnology, Santa Cruz, CA),<br>neurotrophin-3 (NT-3, Santa Cruz Biotechnology, Santa Cruz, CA),<br>nerve growth factor (NGF, Santa Cruz Biotechnology, Santa Cruz, CA),<br>extracellular signal-regulated kinase (ERK; Santa Cruz Biotechnology, Santa Cruz, CA)<br>GAPDH (Santa Cruz Biotechnology, Santa Cruz, CA, USA)
Validation	These antibodies were validated for the species and application on the manufacturer's website.

Eukaryotic cell lines

Policy information about cell lines

Cell line source(s)	Human Schwann Cells were purchased from ScienCell Research Laboratories (catalog no.1700; Carlsbad, CA)
Authentication	Human Schwann Cells from ScienCell Research Laboratories are cryopreserved at passage one and delivered frozen. Each vial contains >5 x 10^5 cells in 1 ml volume. Human Schwann Cells are characterized by immunofluorescence with antibodies specific to S100.
Mycoplasma contamination	Human Schwann Cells are negative for mycoplasma, HIV-1, HBV, HCV, and bacteria.

Commonly misidentified lines (See ICLAC register)	There is no commonly misidentified cell lines in this study.

Animals and other organisms

Policy information about studies involving animals; ARRIVE guidelines recommended for reporting animal research

Laboratory animals	Female New Zealand white rabbits about 2 kg at 12 weeks of age were used.
Wild animals	This study did not involve wild animals.
Field-collected samples	This study did not involve samples collected from the field.
Ethics oversight	This study was approved by the Animal Ethics Committee of Inha University Hospital (INHA 18 0503-560) and animal care and experiments were performed in accordance with established institutional animal ethics committee regulations.

Note that full information on the approval of the study protocol must also be provided in the manuscript.

Flow Cytometry

Plots

Confirm that:

☒ The axis labels state the marker and fluorochrome used (e.g. CD4-FITC).

☒ The axis scales are clearly visible. Include numbers along axes only for bottom left plot of group (a 'group' is an analysis of identical markers).

☒ All plots are contour plots with outliers or pseudocolor plots.

☒ A numerical value for number of cells or percentage (with statistics) is provided.

Methodology

Sample preparation	Whole blood was drawn from the heart of a Sprague-Dawley rat using a disposable syringe containing 2.2 % sodium citrate (9:1, v/v). Rat platelets were isolated from citrated blood with centrifugation method and platelet numbers were adjusted to 4-4.5 x 108/mL for further study. Platelets were stained with isotype control (mouse IgG; Santa Cruz; 1:500) and platelet activation marker (CD62P; SantaCruz, 1:500), followed by incubation in secondary antibody-PE (Invitrogen; 1:1000).
Instrument	CytoFlex Flow Cytometer (Beckman Coulter Life Science)
Software	The data was collected and analyzed using CytExpert program (Beckman Coulter Life Science).
Cell population abundance	N/A
Gating strategy	Control stains [isotype (designated as a negative control) or CD62P (designated as a positive control) stained platelets] were used to set gates. All samples were then FSC-A and SSC-A gated, followed by FSC-A/FSC-H gating to select singlet cells. Subsequent relevant gating was conducted and then the percentage of platelets expressing the analyzed antigens were shown.

☐ Tick this box to confirm that a figure exemplifying the gating strategy is provided in the Supplementary Information.
